# Geographical inequalities in the decreasing 28-day mortality following incident acute myocardial infarction: a Danish register-based cohort study, 1987–2016

**DOI:** 10.1186/s12872-022-02519-7

**Published:** 2022-03-04

**Authors:** Niels Asp Fuglsang, Elisabeth Zinck, Annette Kjær Ersbøll, Bjarne Kjær Ersbøll, Gunnar Hilmar Gislason, Thora Majlund Kjærulff, Kristine Bihrmann

**Affiliations:** 1 DTU Compute, Technical University of Denmark, Kgs Lyngby, Denmark; 2 National Institute of Public Health, University of Southern Denmark, Studiestræde 6, 1455 Copenhagen, Denmark; 3 Department of Cardiology, The Cardiovascular Research Centre, Copenhagen University Hospital Herlev and Gentofte, Gentofte, Denmark; 4 Faculty of Health and Medical Sciences, University of Copenhagen, Copenhagen, Denmark; 5 The Danish Heart Foundation, Copenhagen, Denmark

**Keywords:** AMI, Geographical variation, Epidemiology, Nationwide registers, 28-day mortality, Spatio-temporal analysis

## Abstract

**Background:**

Mortality following acute myocardial infarction (AMI) has decreased in western countries for decades; however, it remains unknown whether the decrease is distributed equally across the population independently of residential location. This study investigated whether the observed decreasing 28-day mortality following an incident AMI in Denmark from 1987 to 2016 varied geographically at municipality level after accounting for sociodemographic characteristics.

**Methods:**

A register-based cohort study design was used to investigate 28-day mortality among individuals with an incident AMI. Global spatial autocorrelation (within sub-periods) was analysed at municipality level using Moran's I. Analysis of spatio-temporal autocorrelation before and after adjusting for sociodemographic characteristics was performed using logistic regression and conditional autoregressive models with inference in a Bayesian setting.

**Results:**

In total, 368,839 individuals with incident AMI were registered between 1987 and 2016 in Denmark; 128,957 incident AMIs were fatal. The 28-day mortality decreased over time at national level with an odds ratio of 0.788 (95% credible interval (0.784, 0.792)) per 5-year period after adjusting for sociodemographic characteristics. The decrease in the 28-day mortality was geographically unequally distributed across the country and in a geographical region in northern Jutland, the 28-day mortality decreased significantly slower (4–12%) than at national level.

**Conclusions:**

During the period from 1987 to 2016, the 28-day mortality following an incident AMI decreased substantially in Denmark. However, in a local geographical region, the 28-day mortality decreased significantly slower than in the rest of the country both before and after adjusting for sociodemographic differences. Efforts should be made to keep geographical trend inequalities in the 28-day mortality to a minimum.

**Supplementary Information:**

The online version contains supplementary material available at 10.1186/s12872-022-02519-7.

## Introduction

Acute myocardial infarction (AMI) is a serious disease causing many deaths both worldwide and in Denmark. Post-AMI survival has significantly improved in western countries over the last decades largely due to substantial improvements in the prophylaxis and treatment of the disease [1–5]. Major changes have taken place during the past 30 years in the Danish healthcare system that have improved the prevention of post-AMI deaths, e.g., increased use of secondary prevention by statins and antithrombotic treatment [6] and enhanced invasive care at fewer specialized hospitals [7]. Even though these changes were implemented at the national level, they could have influenced post-AMI survival differently across the country.

A strong decline in the post-AMI mortality has been shown from 1984 through 2008 at a national level in Denmark [1]. However, studies have shown, that not all population groups seem to benefit equally from the improved prevention and treatment initiatives [6, 8, 9]. In addition, it has been shown that geographical differences in the 28-day mortality following an incident AMI in Denmark exists [10, 11], and only part of these geographical differences can be explained by the geographical variations in sociodemographic characteristics. Hence, it remains unknown if the decline in post-AMI mortality is geographically equally distributed across the country or whether the decline lacks behind in some areas and population groups.

The aim of the present study was to investigate whether the observed decreasing 28-day mortality following an incident AMI in Denmark from 1987 to 2016 varied geographically at municipality level after accounting for sociodemographic characteristics.

## Methods

### Study design and AMI population

The present study was a register-based cohort study including adults with an incident AMI (age $\ge$ 30 years) with residential location in Denmark at the time of AMI in the study period January 1st, 1987 to December 31st, 2016. The outcome was 28-day mortality defined as the proportion of individuals with an incident AMI who died at any time between the day of AMI and 28 days after discharge from the hospital.

Individuals with AMI were identified by International Classification of Disease 8th version (ICD-8) code 410 until end 1993 and 10th version (ICD-10) code I21 from 1994 onwards as primary or secondary diagnosis in the Danish National Patients Register (NPR) or underlying or contributing cause of death registered in the Danish Register of Causes of Death (RCD). All individuals with AMI in the period 1987–2016 were extracted from the NPR and RCD. Furthermore, individuals with previously registered AMI (1977–1986) were excluded in order to include only incident AMI cases in the study period.

The study period, 1987–2016, was divided into six 5-year time periods in order to investigate the development over time while still maintaining enough data within each time period. The AMI data were aggregated at municipality level. The average age of the individuals at date of AMI (denoted AMI age) was calculated within each time period and municipality.

The validity of the AMI diagnosis in the NPR and RCD has previously been reported to be high [12]. However, in Denmark the autopsy rate is below 10% overall, and the mortality statistics of the RCD are not regularly validated [13]. In order to account for the potentially higher uncertainty on AMI diagnoses only registered in the RCD, a supplementary analysis was performed based only on individuals with an AMI diagnosis registered in the NPR.

### Study area

The study area consisted of Denmark (43,000 km^2^), where the healthcare system is tax-financed and access to most primary and secondary healthcare is free-of-charge [14].

In 2007, a reform transferred the primary responsibility for general practitioners and hospitals to 5 newly established administrative regions and the existing 271 municipalities were merged into 98 [14]. In this study, residential locations from 1987 to 2006 were converted into the current 98 municipalities.

In the last time period, 2012–2016, less than 100 incident AMIs occurred in the population of each of the four small island municipalities Fanø, Samsø, Læsø and Ærø (locations shown in Additional file 1: Fig. S1). Therefore, these four municipalities were excluded to ensure the stability of the analysis. The excluded municipalities corresponded to 0.44% of the individuals with AMI and 0.62% of the individuals with a fatal AMI.

### Background population

The background population was defined as the residents in the municipalities in Denmark after the four small island municipalities were excluded. Characteristics of the background population at municipality-level were calculated for each of the six time periods based on the population in each of the five years within each time period.

The municipality-level sociodemographic characteristics were [15]:

1. “Cohabitation”: proportion of adults (individuals ≥ 15 years) who were married or in a registered partnership,
2. “Low education”: proportion of adults with low educational level ($\le$ 9 years),
3. “Unemployment”: the proportion of unemployed among adults (unemployed 6 months or more within a year),
4. “Low income”: the proportion of adults in the lowest third of family equalized income (derived within gender and age groups (15–29, 30–44, 45–64, ≥ 65 years); the cut-off value for the lowest third was based on country level data for every combination of age group and gender).

All variables were standardized within each time period by subtracting the average and dividing by the standard deviation. This was done in order to reflect differences between the municipalities rather than temporal trends that may correlate with the 28-day mortality at a national level. Only a low degree of collinearity between the municipality-level sociodemographic characteristics was observed based on Pearson’s correlation coefficients.

### Statistical analysis

First, 28-day mortality in the municipalities was modelled using a simple logistic regression model with time period (mean centred) as the only covariate in order to investigate geographical inequalities in the 28-day mortality adjusting for a national temporal trend (Model 1).

Second, Model 1 was extended by including AMI age, and municipality-level sociodemographic characteristics: low education, low income, unemployment, and cohabitation (giving Model 2). Linearity of the covariates was evaluated by visual inspection of parameter estimates of a categorical version of the variables. The residuals for each of the six time periods were mapped (on the odds ratio (OR) scale) to visualize the geographical differences in observed and predicted value after adjusting for sociodemographic characteristics.

Data are said to be positively spatially autocorrelated when data points that are geographically closer to one another are more similar than data points further apart. After accounting for sociodemographic characteristics in a logistic regression, spatial structure of the residuals may remain. This is possibly due to an unknown or unmeasured spatially autocorrelated covariate, or because subjects tend to be close to similar subjects (neighbourhood effects) [16]. In order to model spatial autocorrelation in the residuals, Model 2 was extended by adding two spatially autocorrelated random effects denoted the spatial structure component and the temporal structure component (giving Model 3). This extension is similar to that proposed by Bernardinelli et al. (1995) [17] and represents the spatio-temporal pattern in mortality with spatially varying time trends. The spatial and temporal components were assumed to be independent, and both were modelled by the conditional autoregressive (CAR) model proposed by Leroux et al. (2000) [18]. Spatial closeness between the municipalities was defined with a binary adjacency matrix in which entries were 1 if the municipalities shared a border and 0 otherwise. Island municipalities were considered adjacent to a municipality if the two were connected by ferry or bridge.

The spatial and temporal structure components reflect the mortality and trend over time, respectively, for each municipality relative to the national mean after adjustment for sociodemographic characteristics. The ORs of the spatial and temporal structure components were mapped to visualize the geographical inequalities after adjusting for sociodemographic characteristics.

Model 3 was implemented in a Bayesian setting with inference based on Markov chain Monte Carlo (MCMC) simulations using the function ST.CARlinear() from the R-package CARBayesST (version 3.0.1) [19]. The model was estimated using 20,000 samples based on a total of 1,050,000 samples with a burn-in of 50,000 and thinning to every 50th sample. Convergence was assessed by inspection of trace-plots and Geweke’s diagnostic [20]. The random effect variances of the CAR model were assigned inverse-gamma prior distributions with default parameters (1; 0.01) and the fixed effects (effect of time period and sociodemographic characteristics) were assigned the default Gaussian (0; 100,000) prior distribution. The effect of the prior distributions was explored by comparing the obtained parameter estimates with estimates from models with different priors (inverse-gamma distribution with parameters (0.5; 0.005) for the variance of the random effects and Gaussian (0; 200,000) distribution for the fixed effects).

Moran’s I [21] was used to measure the degree of global spatial autocorrelation in the residuals of Models 1, 2 and 3. Monte Carlo simulation with 9999 permutations was used to determine significance of the statistic.

Data management was performed in SAS software version 9.4 and statistical analyses were done in R version 3.6.1 [22].

## Results

**Fig. 1:**
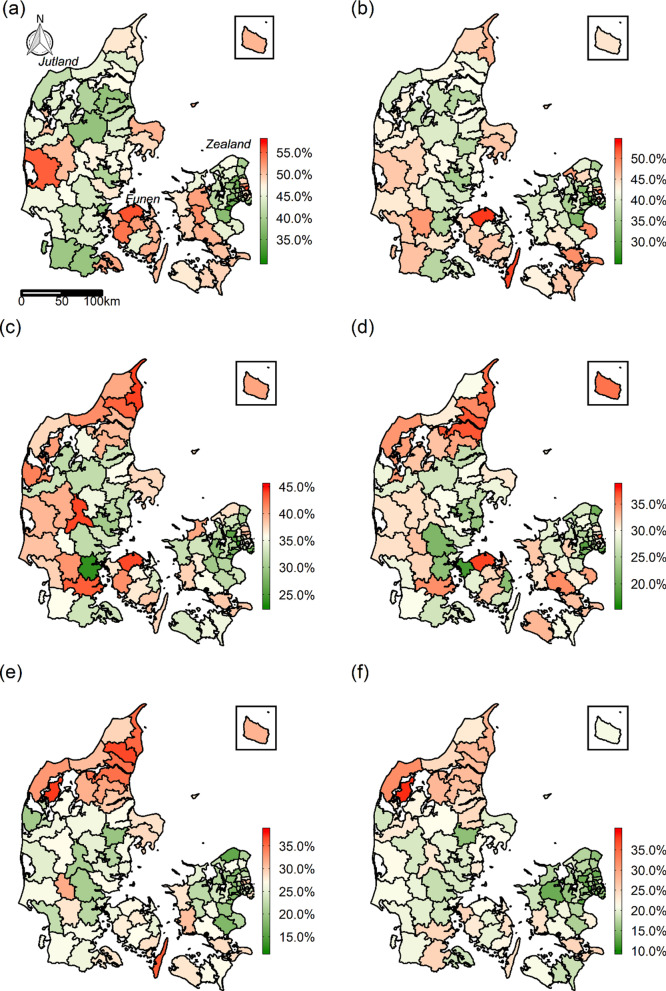
The unadjusted 28-day mortality at municipality level throughout the study period **a** 1987–1991, **b** 1992–1996, **c** 1997–2001, **d** 2002–2006, **e** 2007–2011, **f** 2012–2016. Note that the scales are different for each period due to the decrease in the national mean throughout the study period. The white colour encapsulates the mean across the 94 municipalities in each period. Thus, red colour indicates 28-day mortality above the national mean, and green colour indicates 28-day mortality below the national mean. Data on administrative boundaries were obtained from the Danish Agency for Data Supply and Efficiency

From 1987 to 2016, a total of 368,839 individuals with incident AMI were registered out of which 128,957 (35.0%) died between day of incident diagnosis and 28 days after discharge from hospital. An illustration of the construction of the study population is shown in Additional file 1: Fig. S2. The number of individuals with AMI decreased from 85,146 in 1987–1991 to 45,324 in 2012–2016, and the 28-day mortality decreased from 46.4 to 20.1% during the same time span (Table 1). Relative to the national mean, however, 28-day mortality increased in especially northern Jutland throughout the study period. A decrease was seen on eastern Zealand (Fig. 1). Overall, the mean age at AMI was fairly constant until a slight decrease towards the end of the study period (Table 1). The proportion of men among individuals with AMI increased slightly in 2012–2016.

**Table 1:** Overview of the AMI and background population in the six time periods

	Period					
	1987–1991	1992–1996	1997–2001	2002–2006	2007–2011	2012–2016
AMI population						
Individuals with incident AMI (N)	85,146	69,906	58,408	59,454	50,601	45,324
Deaths within 28 days (N)*	39,518	29,236	20,592	17,342	13,163	9,106
28-day mortality (%)	46.4	41.8	35.3	29.2	26.0	20.1
AMI age (mean (sd))	70.6 (12.7)	70.8 (13.0)	70.5 (13.6)	70.6 (14.0)	70.3 (14.2)	69.3 (13.9)
Men (%)	60.8	59.4	59.2	59.6	60.7	63.0
Background population						
Cohabitation (%)	44.0	43.2	43.0	42.8	42.5	41.4
Low education (%)	48.4	45.0	41.1	38.2	36.4	34.0
Unemployment (%)	3.6	4.6	2.3	2.4	1.1	1.3
Low income (%)	30.6	30.1	30.0	30.2	30.3	31.0

AMI age is the average age of individuals with incident AMI. The characteristics of the background population were calculated as the mean value across the 94 municipalities in the time period

*AMI* acute myocardial infarction, *sd* standard deviation

*Deaths within 28 days is the number of individuals with an incident AMI who died at any time between the day of AMI and 28 days after discharge from the hospital

### Analysis of trends in geographical inequality

In all time periods, Moran’s I showed highly significant positive spatial autocorrelation in the residuals of Model 1, which was only adjusted for the time period (Table 2). This indicated that significant geographical inequality was present in the 28-day mortality displayed in Fig. 1.

**Table 2:** Results of Moran’s I statistic

	Period					
	1987–1991	1992–1996	1997–2001	2002–2006	2007–2011	2012–2016
Model 1	0.43***	0.37***	0.38***	0.27***	0.47***	0.53***
Model 2	0.24***	0.22**	0.10	0.16*	0.13*	0.25***
Model 3	0.13*	0.14*	0.02	0.15*	0.02	0.17**

Moran’s I statistic of the spatial autocorrelation in the residuals in models of increasing complexity. Model 1 adjusted for time period, Model 2 further adjusted for age of individuals at date of AMI, low income, low education, cohabitation and unemployment and Model 3 additionally included spatially autocorrelated random effects. A higher statistic indicates a higher degree of geographical inequality in the data

Significance codes: *P < 0.05, **P < 0.01, ***P < 0.001

**Fig. 2:**
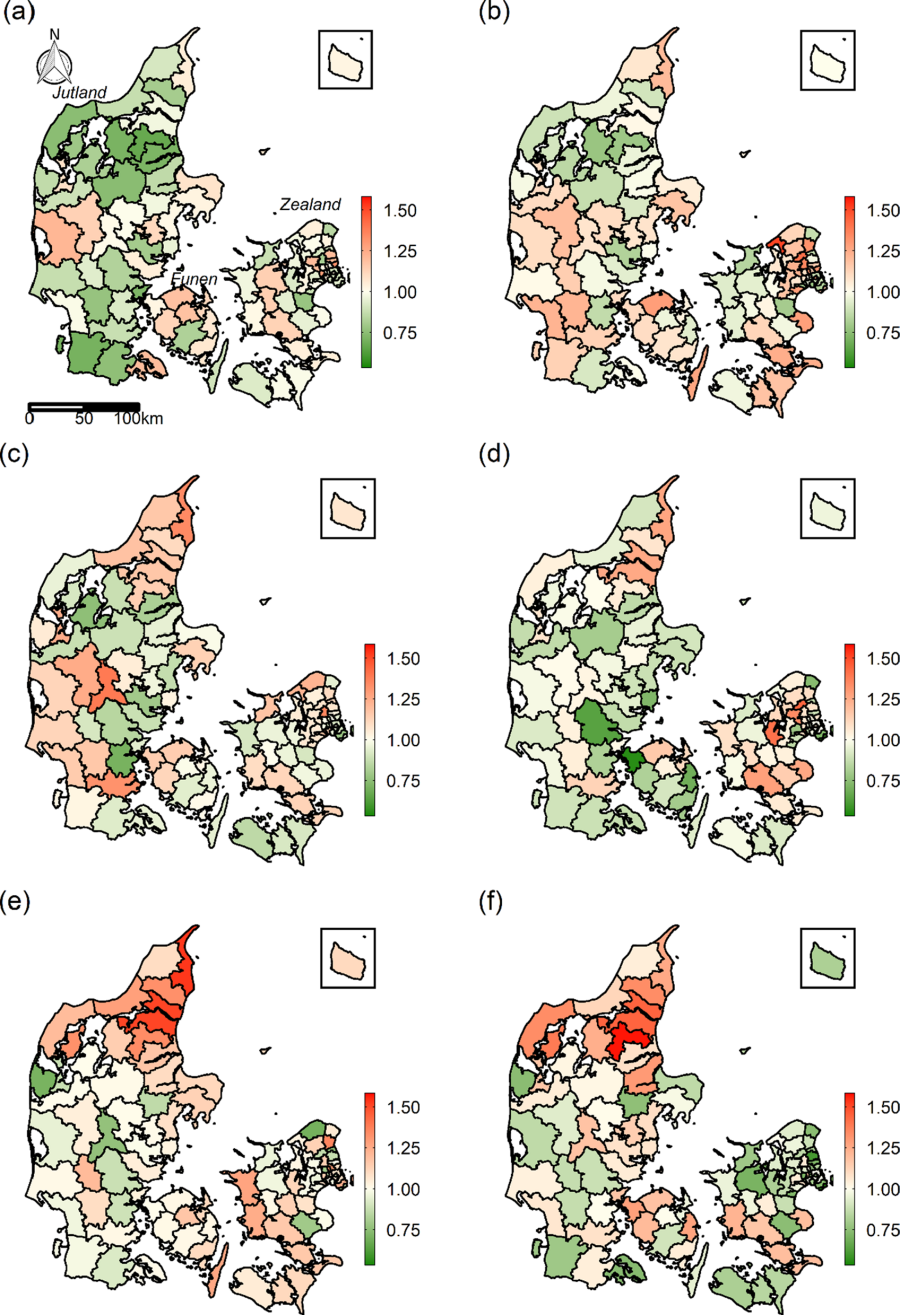
Residual ORs from Model 2 of the 28-day mortality for each time period: **a** 1987–1991, **b** 1992–1996, **c** 1997–2001, **d** 2002–2006, **e** 2007–2011, **f** 2012–2016. The white colour indicates a residual OR of 1 (i.e. the observed mortality was equal to the expected mortality of the model). Thus, red colour indicates the observed mortality was above the expected value, and green colour indicates it was below. Data on administrative boundaries were obtained from the Danish Agency for Data Supply and Efficiency

The residual ORs of Model 2, which was additionally adjusted for sociodemographic characteristics, are visualized in Fig. 2. A residual OR above 1 in a municipality indicates that the 28-day mortality is higher than expected by the model. The residual ORs in northern Jutland increased substantially during the study period and generally lie above 1 in especially the last two time periods. Moran’s I showed significant positive spatial autocorrelation in the residuals of Model 2 in all but the third time period (Table 2). This indicated that significant geographical inequality was still present in the 28-day mortality after adjusting for sociodemographic characteristics.

**Fig. 3:**
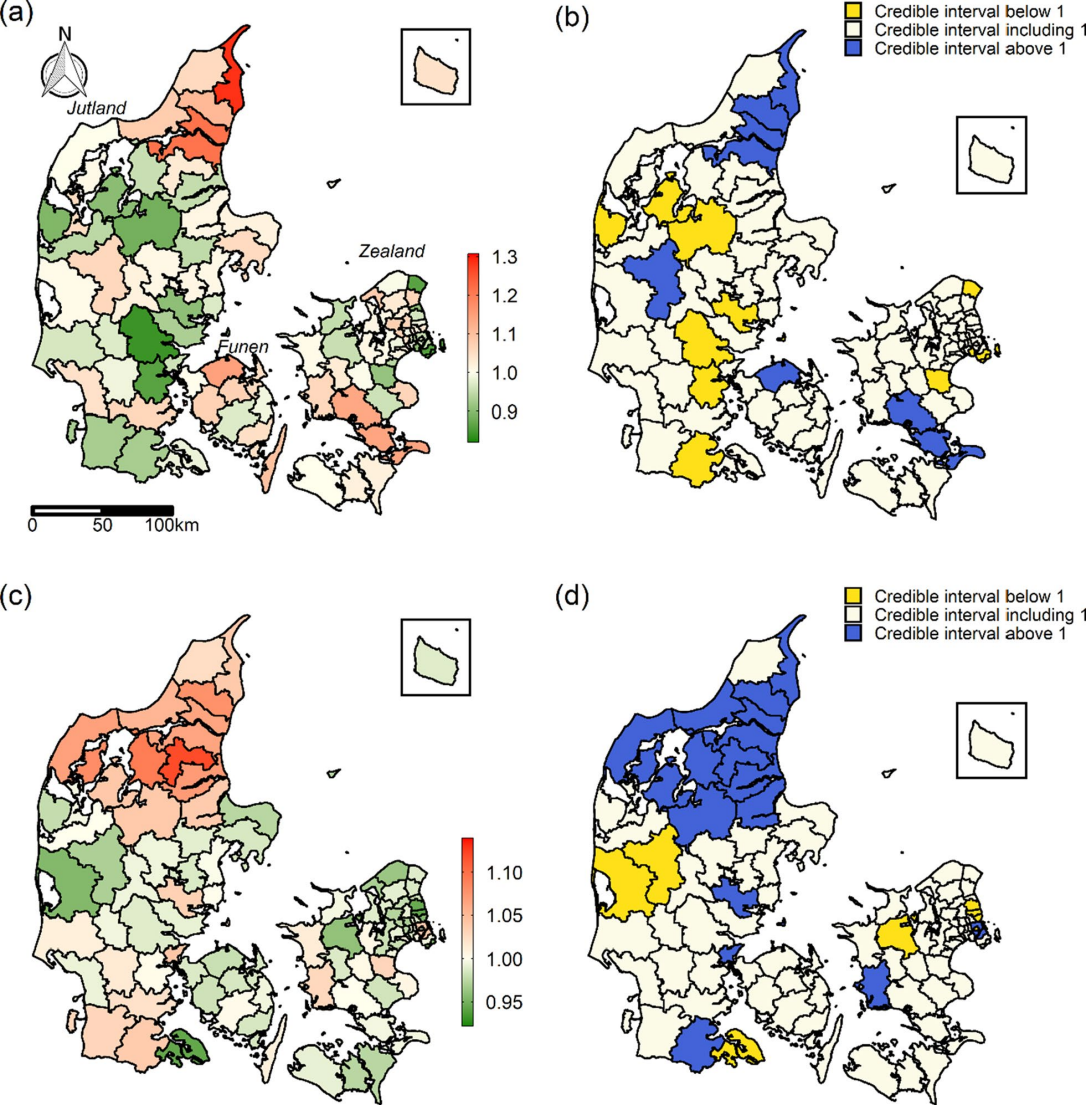
The estimated spatial (**a**) and temporal (**c**) structure component ORs from Model 3. **b** and **d** are certainty maps indicating the span of 95% credible intervals for the spatial and temporal structure component, respectively. For the spatial structure component, red (**a**) and blue (**b**) colour indicates that the 28-day mortality halfway through the study period (since time period was mean centred) was higher than the national mean after adjusting for sociodemographic characteristics, white colour indicates it was similar to the national mean, and green (**a**) and yellow (**b**) colour indicates it was lower. For the temporal structure component, red (**c**) and blue (**d**) colour indicates the 28-day mortality was not decreasing as fast as the national mean after adjusting for sociodemographic characteristics, white colour indicates the trend in 28-day mortality was similar to the national trend, and green (**c**) and yellow (**d**) colour indicates 28-day mortality was decreasing faster than the national mean. Data on administrative boundaries were obtained from the Danish Agency for Data Supply and Efficiency

The estimated spatial and temporal structure component ORs from Model 3 are seen in Fig. 3. The estimated spatial structure component (Fig. 3a) indicates the level of the 28-day mortality in the municipality halfway through the study period (since time period was mean centred) compared to the national mean after adjusting for sociodemographic characteristics. Some geographical structure was seen in the spatial structure component with higher mortality in for example northern Jutland. The estimated temporal structure component indicates how the trend in 28-day mortality in each municipality deviated from the national trend after adjusting for sociodemographic characteristics. The estimated national trend in 28-day mortality was decreasing: OR = 0.788 per 5-year period with 95% credible interval (CI) (0.784, 0.792). This implies that an OR below 1 in the temporal structure component (Fig. 3c) indicates that the 28-day mortality in the municipality was decreasing faster than the national mean after adjusting for sociodemographic characteristics. On the other hand, an OR above 1 indicates that the 28-day mortality was decreasing slower than the national mean. Potentially, the 28-day mortality in a municipality could even be increasing over time, but this was not observed.

The temporal structure component (Fig. 3c) displayed geographical patterns in the decreasing 28-day mortality. ORs above 1 were estimated in all municipalities located in northern Jutland suggesting that the 28-day mortality in this region was not decreasing as fast as in the rest of the country. At municipality level, the estimated decrease in northern Jutland was 4% (95% CI:(1%, 6%)) to 12% (95% CI: (7%, 17%)) slower than the overall decrease at national level.

After the spatially autocorrelated random effects were added to Model 3, the spatial autocorrelation in the residuals was further reduced in all time periods compared to Model 2, but significant spatial autocorrelation remained in the residuals in four out of six time periods (Table 2).

The regression coefficients from Models 1, 2, and 3 are listed in Additional file 2: Table S1. The sensitivity analysis in Model 3 showed that changing the priors of the model did not influence the results.

### Supplementary analysis

A proportion of 18.8% of the AMI cases included in the study were only registered in the RCD (i.e., the AMI diagnosis was not given at a hospital). A supplementary analysis was performed including only those with an AMI diagnosis registered in the NPR using the same models as in the main analysis (tables and figures included in Additional file 3). The supplementary analysis found similar results when excluding individuals registered only in the RCD. In accordance with results from the main analysis, significant geographical inequality was still present in the 28-day mortality in all time periods and adjusting for sociodemographic characteristics could only explain the inequality in two out of six time periods. Adjusted for sociodemographic characteristics, municipalities in northern Jutland, southern Zealand, and the islands south of Zealand had a higher 28-day mortality than the national mean. Additionally, the 28-day mortality also decreased slower in northern Jutland than in the rest of the country in this analysis in line with results from the main analysis. However, in contrast to the main analysis, the geographical effect in Model 3 explained all the geographical inequality in the 28-day mortality when the individuals only registered in the RCD were excluded from the analysis.

The regression coefficients from the supplementary analysis are listed in Additional file 2: Table S2.

## Discussion

### Main findings

This study investigated the geographical patterns in post-AMI mortality among individuals with incident AMI in Denmark in the period 1987 to 2016. Results showed that the 28-day mortality of AMI decreased substantially in 1987–2016, but large differences between municipalities were observed. After adjusting for sociodemographic characteristics, the 28-day mortality decreased more slowly in northern Jutland than in the rest of the country.

### Interpretation of results

The drivers behind the overall decline in AMI mortality rates during the past decades are not fully understood as many potential determinants, including risk factors, treatment, and sociodemographic profile of the population, have been changing simultaneously.

However, trends in AMI mortality have most likely benefited from a decline in risk factors, more effective pharmacological treatments, and better medical and invasive care [23]. In Denmark, five specialized heart centres were established during the study period, a ‘heart programme’ was introduced in 1993 to improve cardiac care and the Danish Heart registry was established in 1998 [9, 24]. These initiatives have almost certainly contributed to the decline in post-AMI mortality. However, this study found a slower decline in AMI mortality in parts of the country compared to the national mean. These geographical inequalities observed after accounting for sociodemographic characteristics might be explained by geographically unequal implementation of treatment standards, distribution of health services, use of preventive treatment or health behaviour across the country.

### Comparisons with other studies

Changes in the geographical inequality of the 28-day mortality over time have not been studied previously in Denmark. Previous work has showed a substantial nationwide decline in post-AMI mortality in Denmark in 1984–2008 [1]. The present study added to these findings by showing that the observed declining nationwide trends in post-AMI mortality vary at municipality level. This finding is important in order to identify health disparities across the country.

A recent Danish study identified a higher AMI mortality in northern Jutland in the years 2005–2014 similar to those found in the second half of the study period in the present study [11]. However, the immediate case fatality and mortality between days 1 and 28 after AMI were analysed separately, and the study additionally adjusted for individual level socioeconomic position which makes it difficult to compare the results to the present study.

A study in the US [25] found that the age standardized decrease of ischemic heart disease mortality 1980–2014 varied significantly between counties, consistent with what was found in the present study. A study in Scotland [26] also found persistent socioeconomic and geographical inequalities in immediate case fatality and 1–27-day mortality during the period 1988–2004 and that small significant geographical variations in mortality remained after adjusting for area-level deprivation. Nevertheless, Davies et al. [23] did not probe into the geographical patterns in mortality trends.

In contrast, another study in the US [27] with data from 1999–2013 found that trends in mortality rates of AMI at county level did not depend on income-level and that mortality trends did not vary across the 4 regions (Northeast, West, Midwest, and South) at large. Similarly, a study in England found no consistent geographical patterns in the trends in post-AMI mortality between regions in 2002–2010 [28]. However, the investigated regions in the studies by Spatz et al. [20] and Smolina et al. [21] are large which could have obscured more local geographical differences in the mortality trends.

### Strengths and limitations

Study assets include the use of a long study period with nationwide data consisting of all incident AMIs in the Danish population aged 30 years or older, which minimized the risk of selection bias.

The study included only administrative data, and it was not possible to adjust for lifestyle risk factors such as smoking and diet. However, adjusting for sociodemographic characteristics might have accounted for some of the variation in lifestyle in the municipalities. It was also not possible to include clinical data on AMI and treatment, including differentiating between STEMI and non-STEMI patients.

Information was available on individuals’ residential location at the time of AMI, along with the individuals’ sex and birth-year. Adjustment for sociodemographic characteristics was only possible at municipality level, and therefore the present study only included models at municipality level. However, adjusting for both municipality and individual level covariates would have allowed for more precise adjustment.

Spatial autocorrelation remained in the residuals after adjustment for sociodemographic characteristics and inclusion of the spatially autocorrelated random effects (Model 3). This may be because the included covariates do not sufficiently account for the sociodemographic differences across municipalities. However, it was possible to model all significant geographical inequality in the supplementary analysis by including the random effects (Model 3), which indicated that the spatial autocorrelation in the residuals may be caused by the inclusion of data from the RCD. This could be because the uncertainty of diagnoses only registered in RCD is larger, and the usage of RCD may differ between regions.

### Implications

Identifying geographical patterns in 28-day mortality and the development of these is important in order to identify inequalities in regional health systems and potentially unheeded areas and populations. Previously reported geographical inequality in 28-day mortality was confirmed, and efforts should be made to decrease these inequalities.

Based on the results found in the present study we find it important to investigate the possible causes of the relative increase in the 28-day mortality in northern Jutland. Particularly, one could investigate whether the structural changes which were introduced in 2007 had any effect. Moreover, investigating changes over time in the use of general practitioners between different regions in Denmark could be relevant in understanding the spatiotemporal pattern in 28-day mortality found in the present study. Ersbøll et al. (2016) found that number of contacts to general practitioner was associated with AMI mortality [10]. Furthermore, future studies could investigate how the treatment of AMI in northern Jutland has changed over time compared to other regions of Denmark.

## Conclusion

During the period from 1987 to 2016 the 28-day mortality following an incident AMI decreased substantially in Denmark. However, in a local geographical region (i.e., northern Jutland) the 28-day mortality decreased significantly slower than in the rest of the country both before and after adjusting for sociodemographic differences. Efforts should be made to find the cause of this slow decrease in 28-day mortality and reduce the post-AMI mortality in this region.

## Supplementary Information

**Additional file 1: Fig. S1.** Map of Denmark; **Fig. S2.** Flow chart.

**Additional file 2: Table S1.** Regression parameter estimates; **Table S2.** Regression parameter estimates including only individuals with an AMI diagnosis registered in the National Patients Register.

**Additional file 3: Fig. S1.** Proportion of fatal AMI registered in the Danish Register of Causes of Death that were also registered in the National Patients Register; **Fig. S2.** Residual ORs from Model 2 including only individuals with an AMI diagnosis in the National Patients Register; **Table S1.** Results of Moran’s I statistic including only individuals with an AMI diagnosis in the National Patients Register; **Fig. S3.** Estimated spatial and temporal structure component including only individuals with an AMI diagnosis in the National Patients Register.

## Data Availability

The data that support the findings of this study are available from the National Health Authority and Statistics Denmark, but restrictions apply to the availability of these data, which were used under license for the current study, and so are not publicly available.

## Abbreviations

AMI: Acute myocardial infarction
ICD-8: International Classification of Disease 8th version
ICD-10: International Classification of Disease 10th version
NPR: National Patients Register
RCD: Danish Register of Causes of Death
OR: Odds ratio
CAR: Conditionally autoregressive
MCMC: Markov chain Monte Carlo
CI: Credible interval
