# Supplementary material for: Geographical inequalities in the decreasing 28-day mortality following incident acute myocardial infarction: a Danish register-based cohort study, 1987–2016
Source: BMC Cardiovasc Disord. 2022 Mar 4;22:81. doi: 10.1186/s12872-022-02519-7 (PMC8896282; doi:10.1186/s12872-022-02519-7)
Supplement: Supplementary file 1 — Additional file 1: Fig. S1. Map of Denmark; Fig. S2. Flow chart. [file 12872_2022_2519_MOESM1_ESM.pdf]

Additional Figures

Figure A.1. Map of Denmark.

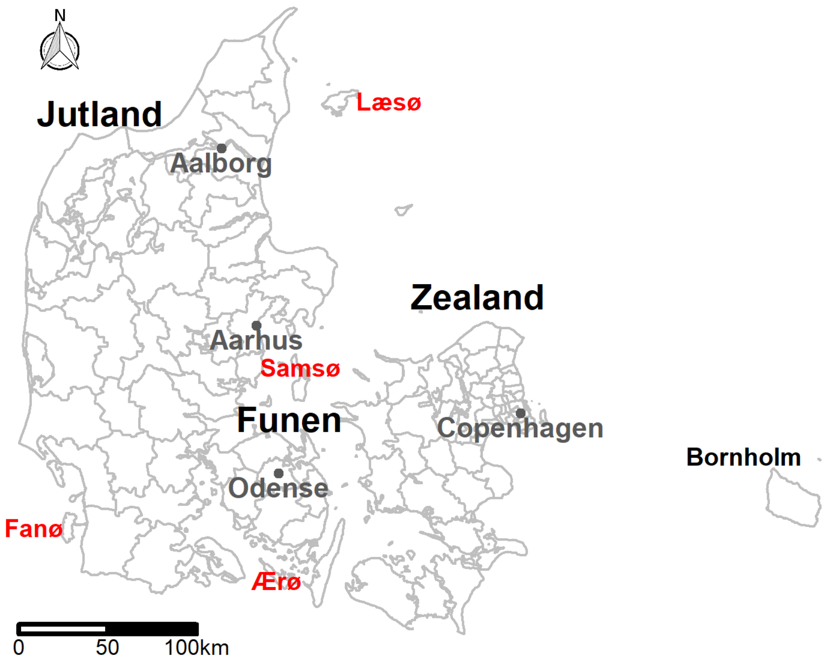

Figure A.1: Map of Denmark with locations mentioned in the analysis. The four island municipalities excluded from the analysis are highlighted in red. Data on administrative boundaries were obtained from the Danish Agency for Data Supply and Efficiency

Figure A.2. Flow chart

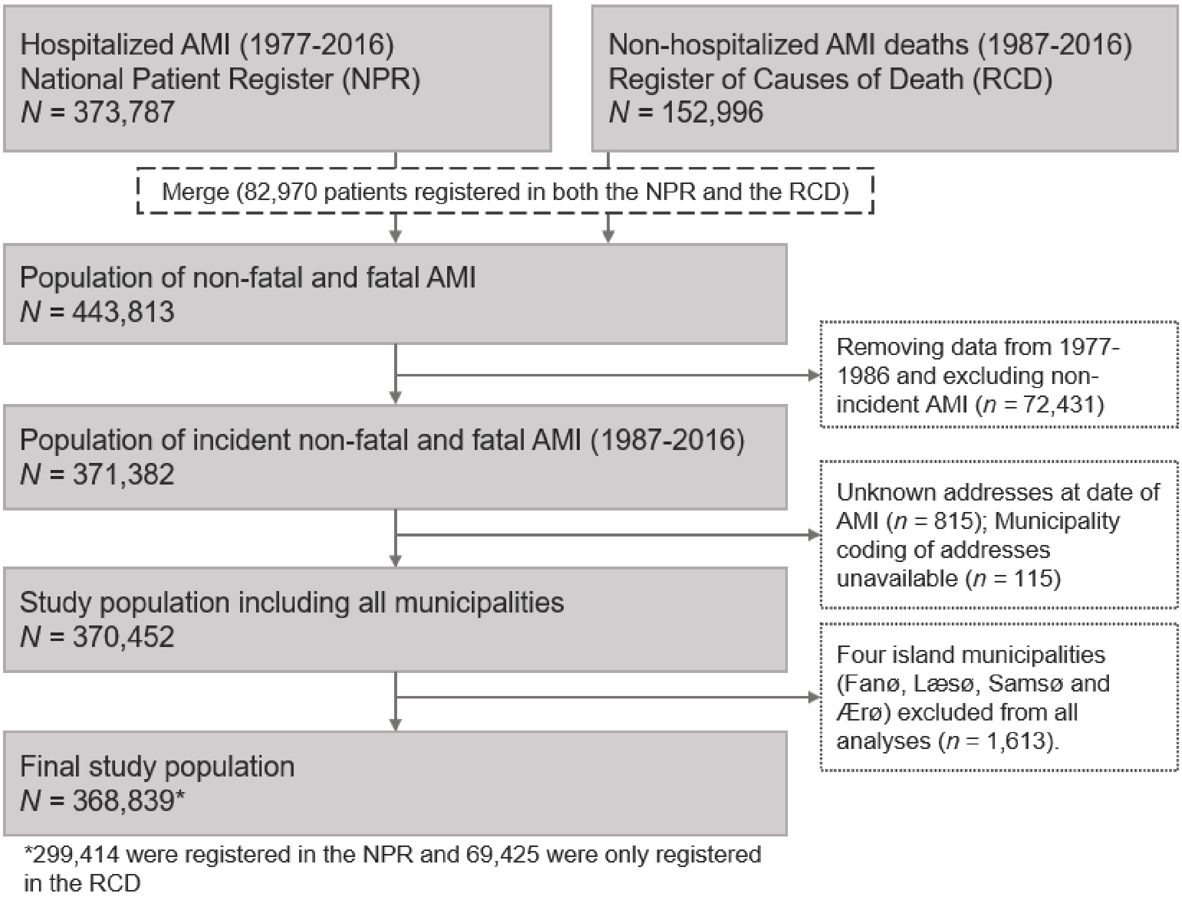

Figure A.2: Flow chart illustrating the construction of the study population based on data from nationwide population registers. AMI, acute myocardial infarction.
