# Supplementary material for: Geographical inequalities in the decreasing 28-day mortality following incident acute myocardial infarction: a Danish register-based cohort study, 1987–2016
Source: BMC Cardiovasc Disord. 2022 Mar 4;22:81. doi: 10.1186/s12872-022-02519-7 (PMC8896282; doi:10.1186/s12872-022-02519-7)
Supplement: Supplementary file 2 — Additional file 2: Table S1. Regression parameter estimates; Table S2. Regression parameter estimates including only individuals with an AMI diagnosis registered in the National Patients Register. [file 12872_2022_2519_MOESM2_ESM.pdf]

Regression coefficients

Table B.1: Regression parameter estimates.

	Model 1	Model 2	Model 3
**Intercept**	-0.77 (-0.79,-0.75)	-0.77 (-0.78,-0.75)	-0.77 (-0.78,-0.76)
**Time period**	-0.24 (-0.26,-0.23)	-0.24 (-0.25,-0.23)	-0.24 (-0.24,-0.23)
**AMI age**	-	0.14 (0.12,0.16)	0.15 (0.13,0.17)
**Unemployment**	-	0.00 (-0.02,0.02)	-0.01 (-0.03,0.00)
**Low education**	-	0.04 (0.01,0.07)	0.06 (0.03,0.10)
**Cohabitation**	-	0.01 (-0.02,0.03)	-0.03 (-0.06,0.00)
**Low income**	-	0.03 (0.00,0.07)	-0.01 (-0.05,0.03)

AMI, acute myocardial infarction

Table B.1: Parameter estimates with 95% confidence (for Model 3 credibility) intervals in the three different models. Note that the covariates have been standardized for each time period and that the estimates are presented in the logit domain.

Table B.2: Regression parameter estimates including only individuals with an AMI diagnosis registered in the National Patients Register.

	Model 1	Model 2	Model 3
**Intercept**	-1.58 (-1.6,-1.56)	-1.58 (-1.59,-1.56)	-1.57 (-1.58,-1.56)
**Time period**	-0.27 (-0.28,-0.26)	-0.27 (-0.28,-0.26)	-0.27 (-0.27,-0.26)
**AMI age**	-	0.13 (0.11,0.15)	0.13 (0.11,0.15)
**Unemployment**	-	-0.01 (-0.02,0.01)	0 .00 (-0.02,0.02)
**Low education**	-	0.04 (0.01,0.07)	0.05 (0.01,0.09)
**Cohabitation**	-	-0.01 (-0.04,0.01)	-0.03 (-0.06,0.01)
**Low income**	-	0.03 (-0.01,0.07)	-0.01 (-0.06,0.03)

AMI, acute myocardial infarction

Table B.2: Parameter estimates with 95% confidence (for Model 3 credibility) intervals in the three different models including only individuals with an AMI diagnosis registered in the National Patients Register. Note that the covariates have been standardized for each time period and that the estimates are presented in the logit domain.
