# Supplementary material for: Geographical inequalities in the decreasing 28-day mortality following incident acute myocardial infarction: a Danish register-based cohort study, 1987–2016
Source: BMC Cardiovasc Disord. 2022 Mar 4;22:81. doi: 10.1186/s12872-022-02519-7 (PMC8896282; doi:10.1186/s12872-022-02519-7)
Supplement: Supplementary file 3 — Additional file 3: Fig. S1. Proportion of fatal AMI registered in the Danish Register of Causes of Death that were also registered in the National Patients Register; Fig. S2. Residual ORs from Model 2 including only individuals with an AMI diagnosis in the National Patients Register; Table S1. Results of Moran’s I statistic including only individuals with an AMI diagnosis in the National Patients Register; Fig. S3. Estimated spatial and temporal structure component including only individuals with an AMI diagnosis in the National Patients Register. [file 12872_2022_2519_MOESM3_ESM.pdf]

Supplementary analysis

Figure C.1. Proportion of fatal AMI registered in RCD that were also registered in the NPR.

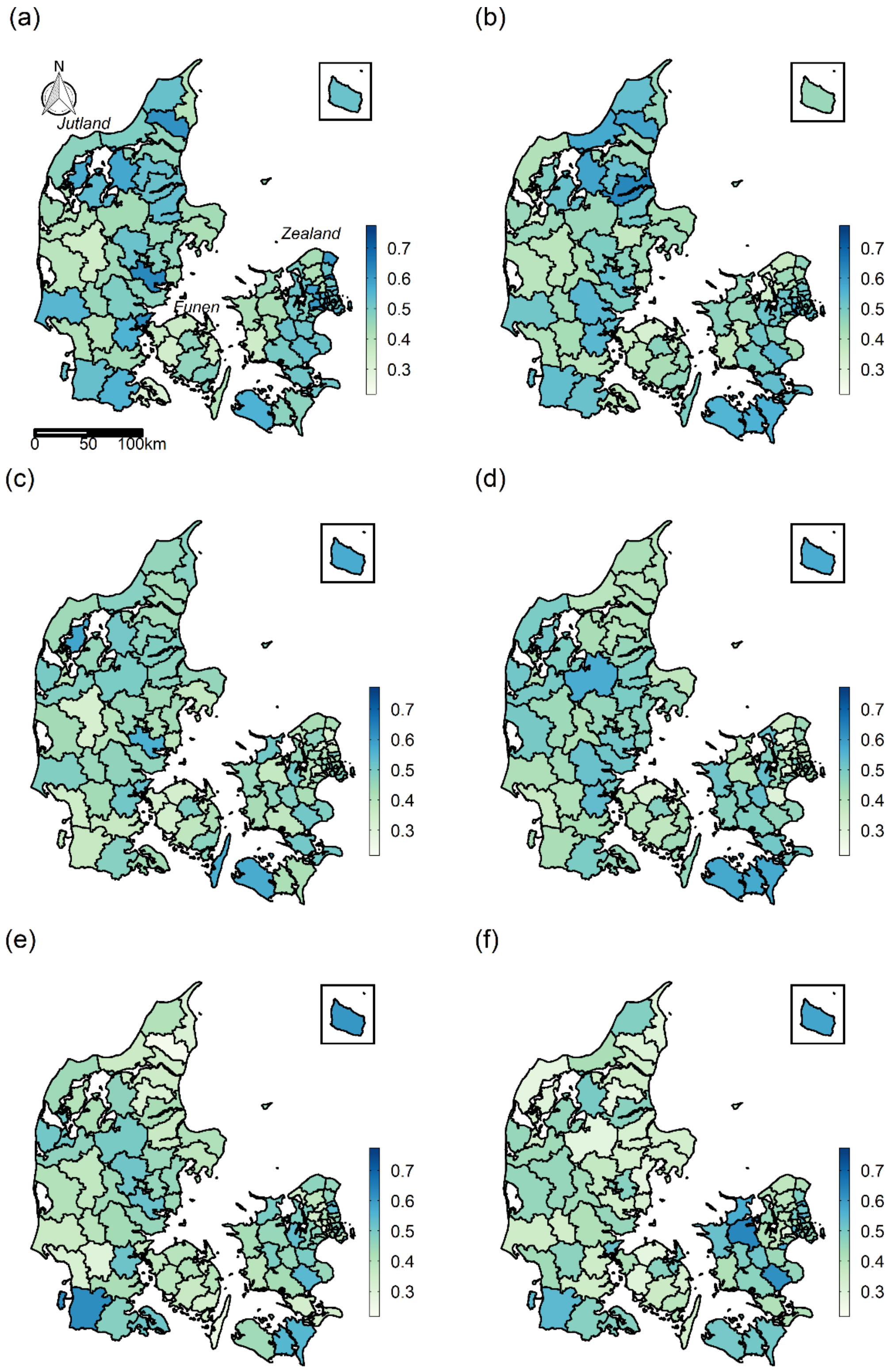

Figure C.1: Proportion of fatal AMI registered in the RCD that were also registered in the NPR in the six time periods: (a) 1987–1991, (b) 1992–1996, (c) 1997–2001, (d) 2002–2006, (e) 2007–2011, (f) 2012–2016. The proportion spans from 24% to 75%. The proportion varies across the municipalities, but no clear patterns were seen. Data on administrative boundaries were obtained from the Danish Agency for Data Supply and Efficiency.

Figure C.2. Residual ORs from Model 2 including only individuals with an AMI diagnosis in the NPR.

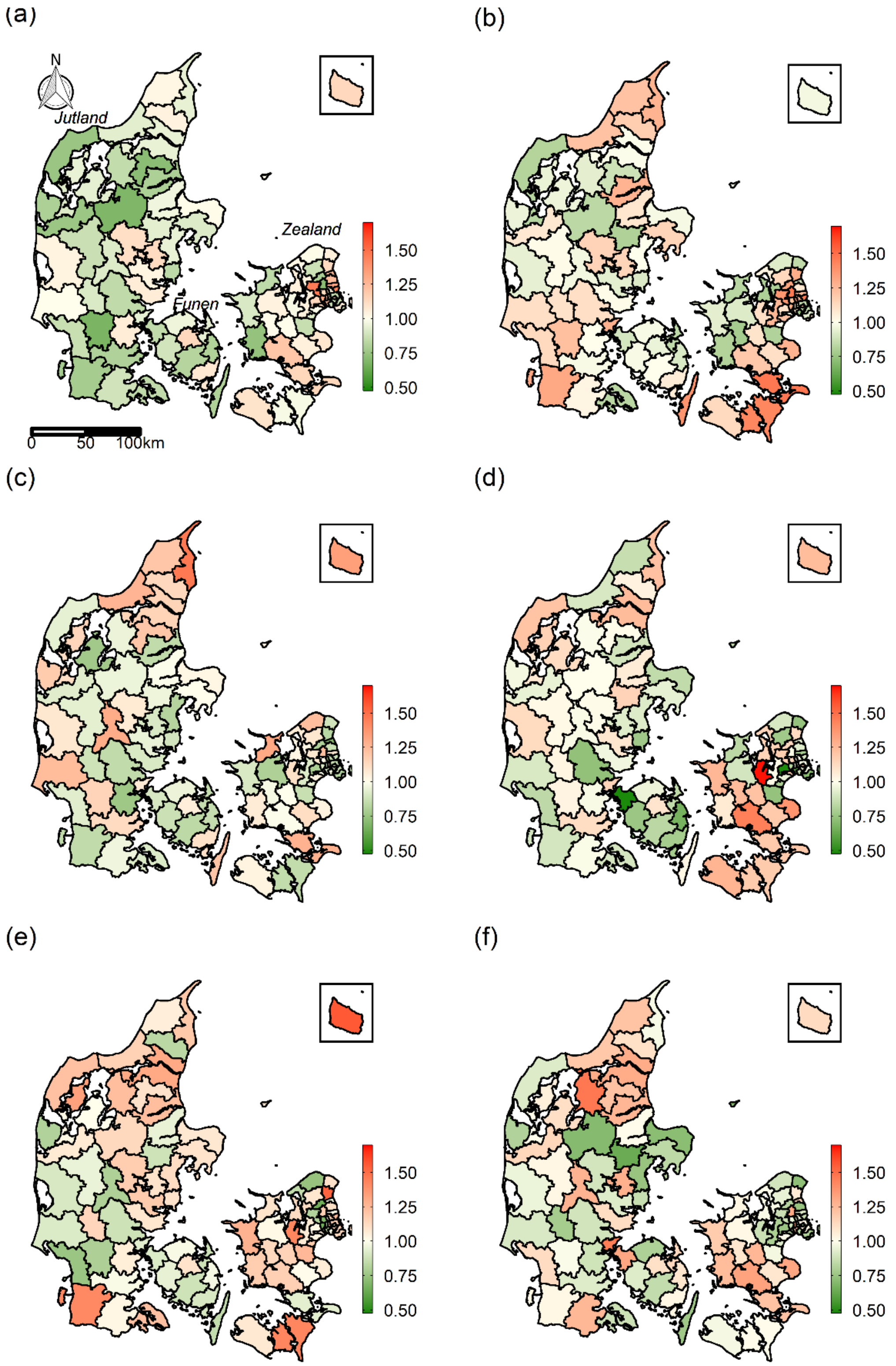

Figure C.2: Residual ORs from Model 2 of the 28-day mortality for each time period: (a) 1987–1991, (b) 1992–1996, (c) 1997–2001, (d) 2002–2006, (e) 2007–2011, (f) 2012–2016. The white colour indicates a residual OR of 1 (i.e. the observed mortality was equal to the expected mortality of the model). Thus, red colour indicates the observed mortality was above the expected value, and green colour indicates it was below. Data on administrative boundaries were obtained from the Danish Agency for Data Supply and Efficiency.

Table C.1: Results of Moran's I statistic including only individuals with an AMI diagnosis in the NPR.

	**Period**					
	1987-1991	1992-1996	1997-2001	2002-2006	2007-2011	2012-2016
**Model 1**	0.20**	0.30***	0.43***	0.39***	0.43***	0.36***
**Model 2**	0.29***	0.20**	0.02	0.09	0.19**	0.12*
**Model 3**	0.10	0.09	0.04	0.05	0.05	-0.04

Significance codes: *P<0.05, **P<0.01, ***P<0.001.

Table C.1: Moran's I statistic of the spatial autocorrelation in the residuals in models of increasing complexity when only including individuals with an AMI diagnosis in the NPR. Model 1 adjusted only for time period, Model 2 further adjusted for age of the individuals at date of AMI, low income, low education, cohabitation and unemployment and Model 3 additionally included spatial effects. Note that Model 3 was able to account for all significant geographical inequality in 28-day mortality.

Figure C.3. Estimated spatial and temporal structure component including only individuals with an AMI diagnosis in NPR.

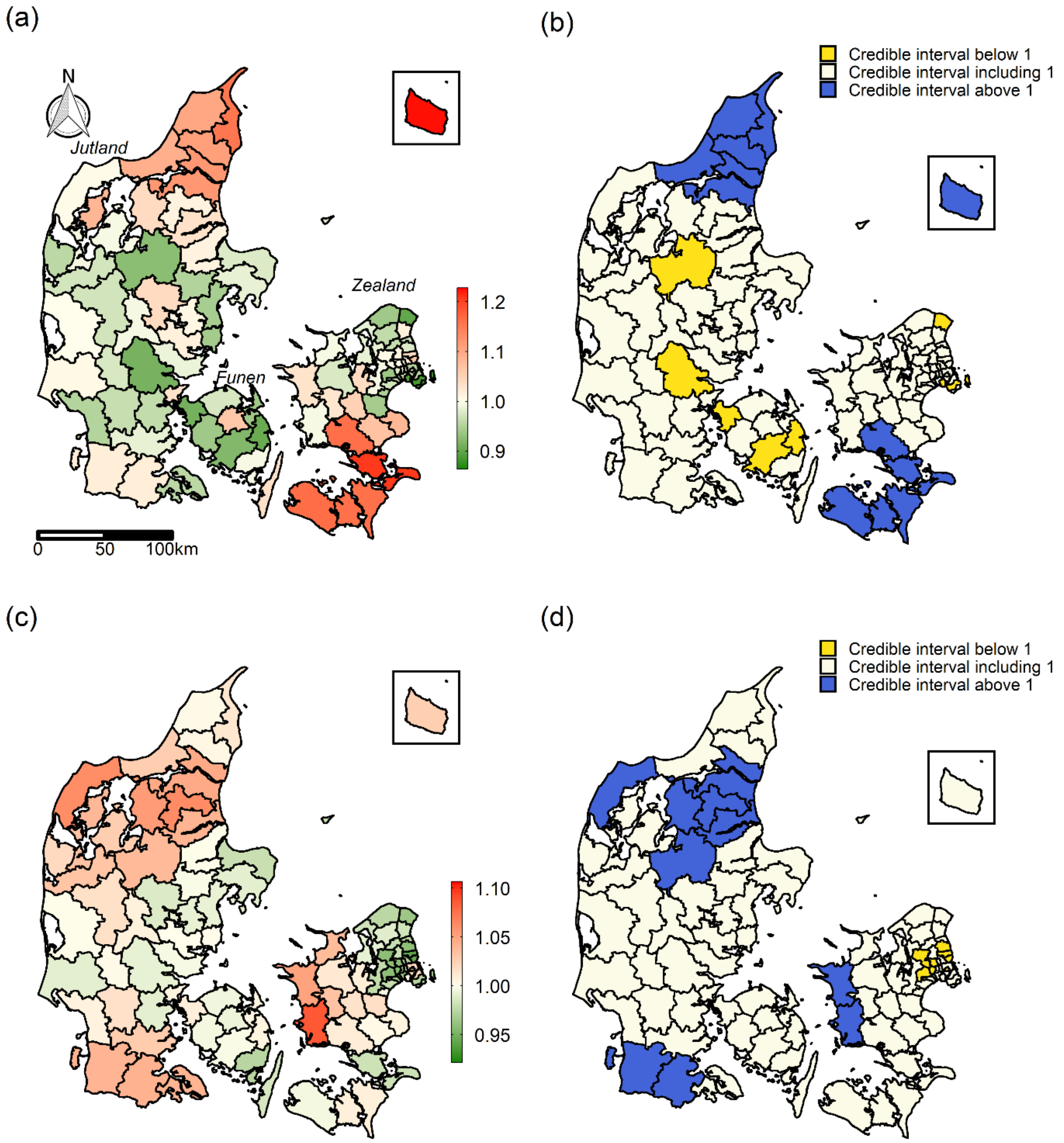

Figure C.3: The estimated spatial (a) and temporal (c) structure component ORs from Model 3. (b) and (d) are certainty maps indicating the span of 95% credible intervals for the spatial and temporal structure component, respectively. For the spatial structure component, red (a) and blue

(b) colour indicates that the 28-day mortality halfway through the study period (since time period was mean centred) was higher than the national mean after adjusting for sociodemographic characteristics, white colour indicates it was similar to the national mean, and green (a) and yellow (b) colour indicates it was lower. For the temporal structure component, red (c) and blue (d) colour indicates the 28-day mortality was not decreasing as fast as the national mean after adjusting for sociodemographic characteristics (white colour indicates the trend in 28-day mortality was similar to the national trend, and green (c) and yellow (d) colour indicates 28-day mortality was decreasing faster than the national mean). Data on administrative boundaries were obtained from the Danish Agency for Data Supply and Efficiency.
